# Supplementary material for: Rectal Epithelial Stem Cell Kinetics in Acute Radiation Proctitis
Source: Int J Mol Sci. 2024 Oct 19;25(20):11252. doi: 10.3390/ijms252011252 (PMC11508457; doi:10.3390/ijms252011252)
Supplement: Supplementary file 1 [file ijms-25-11252-s001.zip › ijms-3162846-supplementary.pdf]

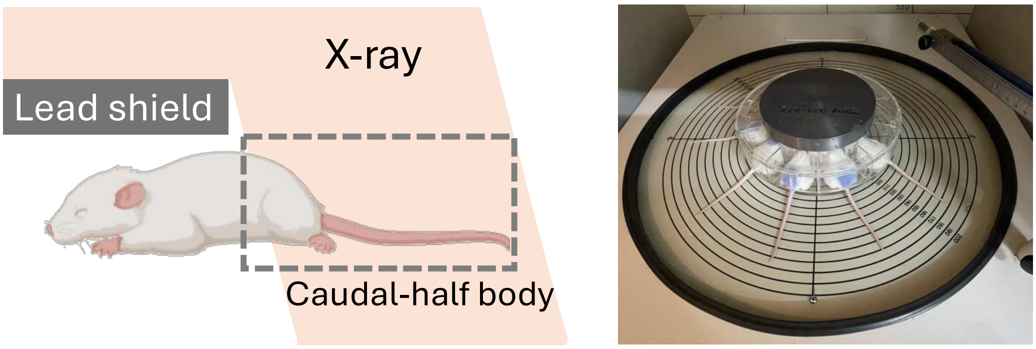

Supplementary Figure S1: Representative images of the CHBI irradiation setup. Caudal half-body irradiation (CHBI).

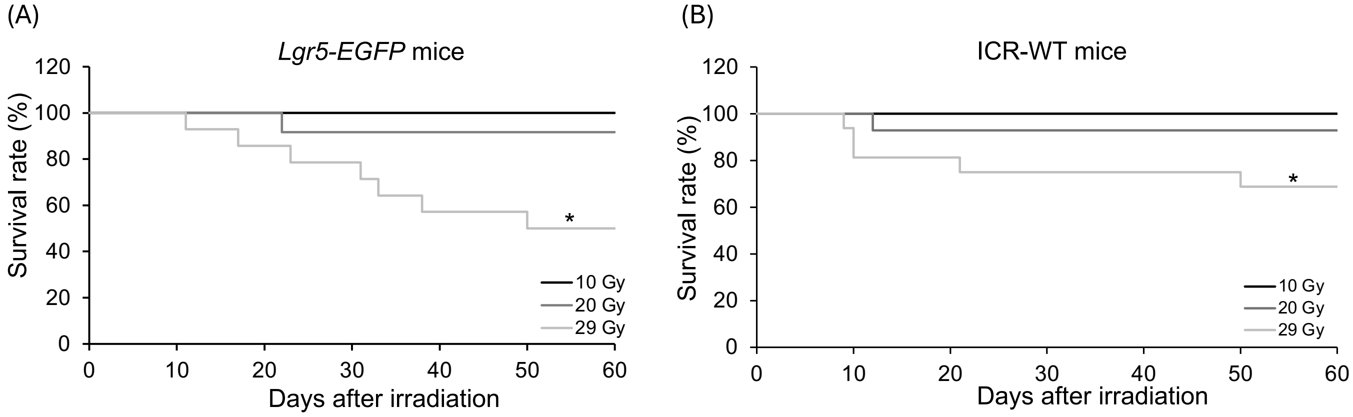

Supplementary Figure S2: 60 days survival test of 10 Gy, 20 Gy and 29 Gy-CHBI irradiated mice (A) Male and Female *Lgr5-EGFP* mice (B) Female ICR wild-type mice. Asterisks indicate a significant difference (*p<0.05) between groups. Caudal half-body irradiation (CHBI); enhanced green fluorescent protein (EGFP); gray (Gy); leucine rich repeat containing G protein-coupled receptor 5 (*Lgr5*).

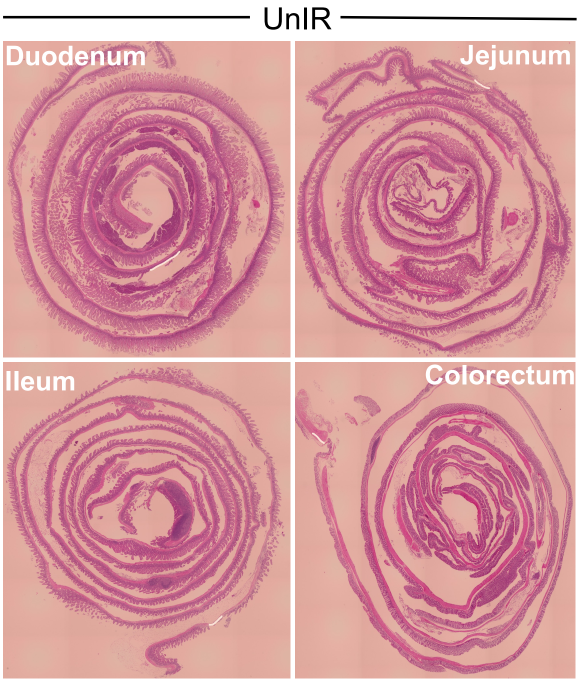

Supplementary Figure S3: Unirradiated group image of the intestine. The solid line underlines the damaged area. Unirradiated (UnIR).

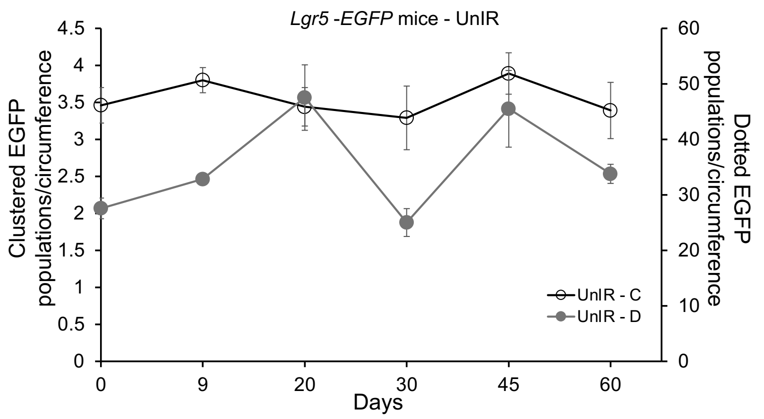

Supplementary Figure S4: 60-day unirradiated group data of *Lgr5-EGFP* mice. Data represent the mean ± standard deviation. Enhanced green fluorescent protein (EGFP); leucine rich repeat containing G protein-coupled receptor 5 (*Lgr5*); unirradiated (UnIR).

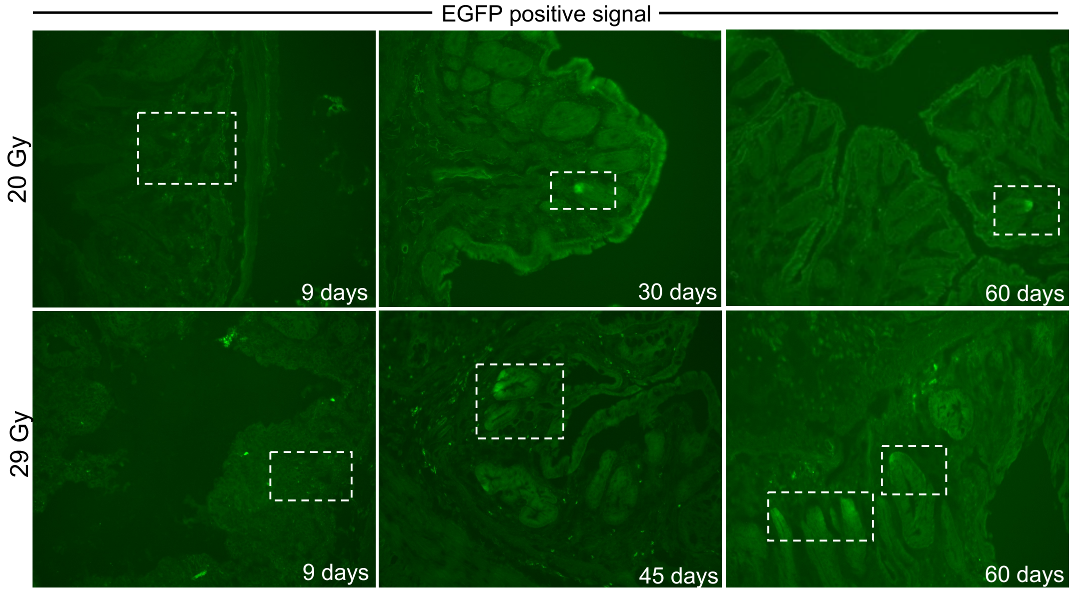

Supplementary Figure S5: Representative images of reappearances of the EGFP population post-irradiation (day 9 to day 60) in 20 Gy (upper panel) and 29 Gy (lower panel) in *Lgr5-EGFP* mice. Magnification 20x. Caudal half-body irradiation (CHBI); enhanced green fluorescent protein (EGFP); gray (Gy); leucine rich repeat containing G protein-coupled receptor 5 (*Lgr5*).

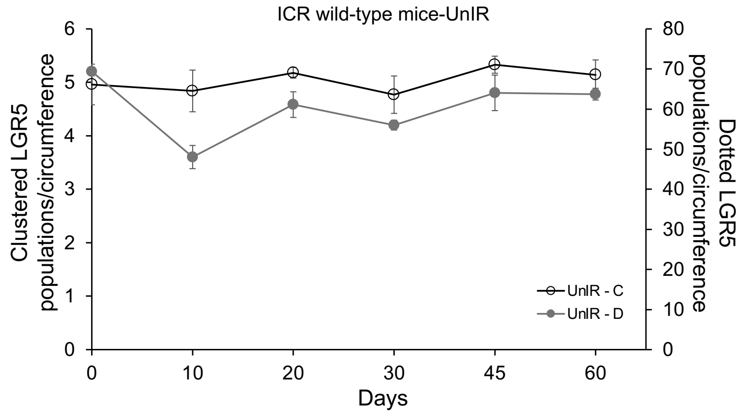

Supplementary Figure S6: 60-day unirradiated group data of wild-type mice. Data represent the mean ± standard deviation. Leucine rich repeat containing G protein-coupled receptor 5 (LGR5); unirradiated (UnIR).

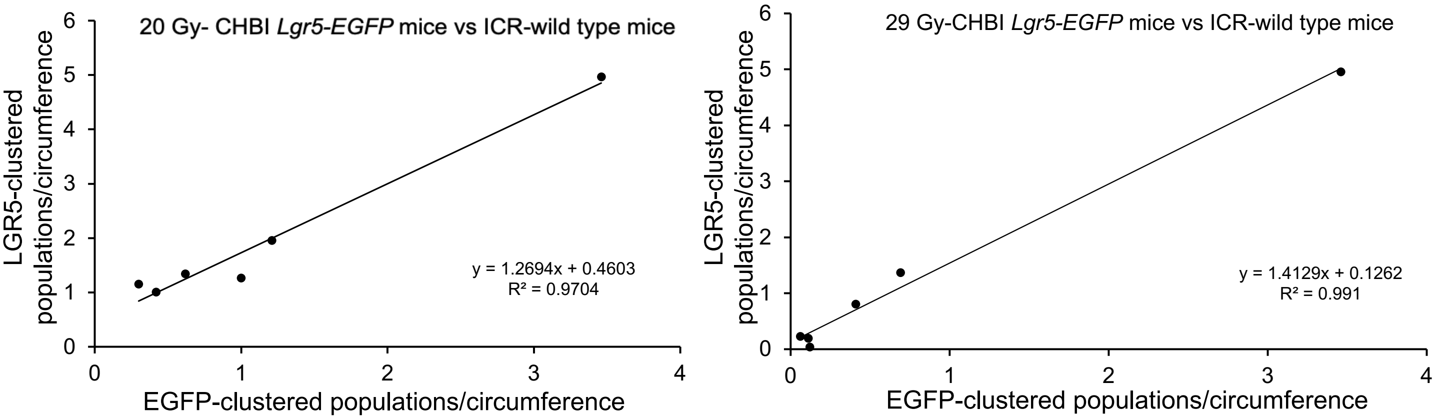

Supplementary Figure S7: Correlation of clustered cell population recovery between *Lgr5-EGFP* mice and ICR-wild type mice in 20 Gy and 29 Gy-CHBI irradiations. Caudal half-body irradiation (CHBI); enhanced green fluorescent protein (EGFP); gray (Gy); leucine rich repeat containing G protein-coupled receptor 5 (*Lgr5*).

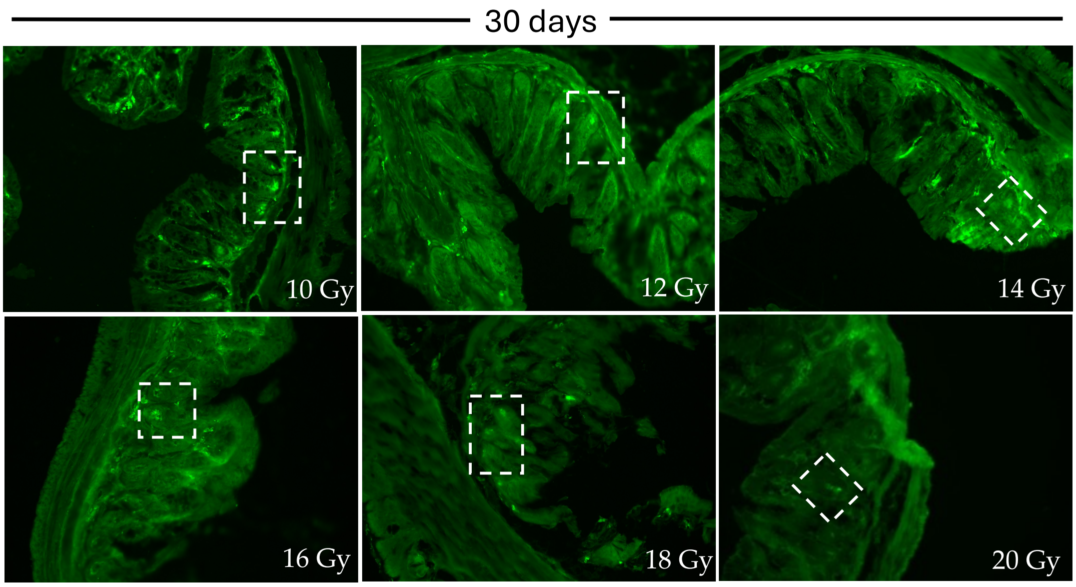

Supplementary Figure S8: Representative images of LGR5 positive clustered stem cell population (Antibody staining) in 10 Gy,12 Gy,16 Gy,18 Gy and 20 Gy irradiated mice. Gray (Gy); leucine rich repeat containing G protein-coupled receptor 5 (LGR5).
